# Bacteriuria and vitamin D deficiency: a cross sectional study of 385 nursing home residents

**DOI:** 10.1186/s12877-019-1400-z

**Published:** 2019-12-30

**Authors:** Rebeka Arnljots, Egill Snaebjörnsson Arnljots, Jörgen Thorn, Marie Elm, Michael Moore, Pär-Daniel Sundvall

**Affiliations:** 1 Research and Development Primary Health Care, Region Västra Götaland, Research and Development Centre Södra Älvsborg Sweden, Sven Eriksonsplatsen 4, SE-503 38 Borås, Sweden; 2 Department of Public Health and Community Medicine/Primary Health Care, Institute of Medicine, Sahlgrenska Academy at the University of Gothenburg, Box 454, SE-405 30 Gothenburg, Sweden; 3 Närhälsan Heimdal Health Care Center, Stengärdsgatan 22, SE-503 34 Borås, Sweden; 4 Närhälsan Fristad Health Care Center, Tärnavägen 6, SE-513 33 Fristad, Borås, Sweden; 5 Health Care Unit Borås Municipality, Ramnåsgatan 1, SE-501 80 Borås, Sweden; 6 Academic Unit of Primary Care and Population Sciences, Faculty of Medicine, University of Southampton, Aldermoor Health Centre, Aldermoor Close, Southampton, SO16 5ST UK; 7 Närhälsan Sandared Health Care Center, Strandvägen 11, SE-518 32 Sandared, Borås, Sweden

**Keywords:** Vitamin D, Bacteriuria, Urinary tract infections, Homes for the aged, Nursing homes, Frail elderly

## Abstract

**Background:**

Up to half of elderly people at nursing homes have asymptomatic bacteriuria, and concentrations of 25-hydroxyvitamin D (25OHD) are generally low. Vitamin D is a modulator of the immune system and involved in protection of the epithelium in the urinary tract as well. The objective was to determine a possible association between bacteriuria and vitamin D deficiency among elderly people at nursing homes.

**Methods:**

Cross-sectional study: Voided urine specimens and blood samples for cultivation and analysis of 25OHD were collected from elderly people at nursing homes in Sweden. Exclusion criteria were: urinary catheter, ongoing antibiotic treatment, incontinence or dementia too severe to provide a voided urine specimen or leave a blood sample, unwillingness to participate or terminal illness. Urine cultures and serum 25OHD concentrations were outcome measures and the association of bacteriuria with vitamin D deficiency was determined by logistic regression.

**Results:**

Twenty-two nursing homes participated and 385 of 901elderly people provided voided urine specimens and blood samples. The mean age was 87 (SD 6.7), 69% women, 19% received vitamin D supplement, 13% had diabetes mellitus, and 54% were diagnosed with dementia. There was significant growth of potentially pathogenic bacteria in 32% (123/385) of voided urine specimens. *Escherichia coli* were present in 83% of positive urine cultures. The mean concentration of 25OHD in serum was 35 nmol/L (SD 21). Thirty-seven per cent (143/385) had 25OHD < 25 nmol/L, and 3.1% (12/385) 25OHD < 12.5 nmol/L. No association between bacteriuria and 25OHD < 25 nmol/L, OR 1.4 (0.86–2.3; *p* = 0.18) adjusted for age, gender, diabetes mellitus and dementia was found. However, if using 25OHD < 12.5 nmol/L as a cut-off for vitamin D deficiency the adjusted odds-ratio was 4.4 (1.1–17; *p* = 0.031).

**Conclusions:**

Bacteriuria and vitamin D deficiency was common. No association between bacteriuria and 25OHD < 25 nmol/L was found. If using 25OHD < 12.5 nmol/L as cut-off for vitamin D deficiency there was an association. However, this has to be interpreted with caution as causality cannot be evaluated as well as only few residents had 25OHD < 12.5 nmol/L.

## Background

Urinary tract infection (UTI) is the most common infection in residents of nursing homes for the elderly. Nearly half the residents will also have asymptomatic bacteriuria (ASB) [1–3]. Differentiating infection from asymptomatic carriage is often difficult leading to inappropriate prescribing of antibiotics. Changes associated with ageing such as multimorbidity, weakening of the immune system and decreased cognitive function, increase the risk of developing UTI [3, 4].

Studies have shown that serum concentrations of vitamin D in elderly individuals are in the lower intervals and even lower among nursing home residents where vitamin D deficiency is common [4–8]. Sweden is at a latitude of 55–69° N, where dermal synthesis of vitamin D occurs solely during the summer months [9]. Despite a lack of consensus on the optimum serum concentrations of vitamin D [10, 11], 25-hydroxyvitamin D (25OHD) ≥ 50 nmol/L is recommended for those > 65 [11].

Vitamin D is a modulator of the immune system, influencing innate and acquired immune reactions, and is involved in protection of the epithelium of the urinary tract [4, 12–14]. The urinary tract is usually considered sterile apart from the urinary meatus [14]. Several protective factors including antimicrobial peptides (AMP) and the innate immune system act to prevent infections of the urinary tract. Vitamin D supports and enhances these systems. The innate immune system is the first-line rapid response barrier to prevent microbial invasion consisting of receptors, proteins and cells that quickly recognize and neutralize foreign bodies. A fast innate immune response is important in preventing the development of UTI, since the adaptive immune system is only later activated. AMPs are synthesized by immune and epithelial cells and offer quick protection by binding foreign microbes and neutralizing them [12, 14, 15].

It has been demonstrated that the most important AMP in the urinary tract is cathelicidin, synthesized by urinary epithelium and released immediately upon exposure to foreign microbes, such as *E. coli*, thus preventing infection [12, 14]. Studies indicate that vitamin D stimulates the production of cathelicidin in the urinary bladder, and there is some evidence supporting positive effects of vitamin D on UTI in children, pre- and postmenopausal women, pregnant women, prediabetic patients and renal transplant patients [12, 16–21]. There is uncertainty concerning the association between vitamin D concentrations and bacteriuria among elderly residents in nursing homes.

Previous studies have shown an increased frequency of symptomatic UTI in those earlier identified with asymptomatic bacteriuria (ASB) and ASB causes a low grade inflammation of the epithelium in the urinary tract [22–24]. As the population ages the burden of ASB and UTI will increase, posing a risk for increased antibiotic use and subsequent resistance to urinary tract antibiotics [3, 4]. Potentially, vitamin D could be a complement in the prevention of ASB and UTI.

The objective was to determine a possible association between bacteriuria and vitamin D deficiency among elderly people at nursing homes.

## Methods

From January to March 2012 in southwestern Sweden (latitude 57.58° N-57.82° N), blood and urine samples were gathered, and a case report form was filed for all included residents of 22 nursing homes. Detailed verbal and written information regarding study procedure was given to the nurses. The Regional ethical review board of Gothenburg University (reference number 578–11) approved the study. Data was collected together with data from other studies [5, 25, 26]. The data presented on Vitamin D and bacteriuria in this manuscript has not been published before.

### Inclusion and exclusion criteria

Inclusion criteria were permanent residency in nursing homes for the elderly, regardless of gender and duration of residency, nursing home resident during the study, approved participation, absence of indwelling urinary catheter, ability to leave a voided urine sample and in case of dementia inclusion only if cooperative when collecting urine and blood samples.

Exclusion criteria were urostomy, terminal illness, intermittent catheterisation, ongoing antibiotic treatment and discontinued study participation.

### Statement of consent

Included residents were provided both written and verbal information. Informed approval was obtained from decision-capable individuals. However, many participants had varying forms of dementia. If a resident lacked comprehension of the provided information, they participated only if they or their surrogates did not refuse participation after the provision of information regarding the study. This procedure was approved by the Regional ethical review board of Gothenburg University (reference number 578–11).

### Case report form

There was a predetermined date for the gathering of blood and urine samples from included residents. Blood and urine samples were collected on the same day. Furthermore, on the same day nurses registered age, gender, vitamin D supplementation, dementia or diabetes, temperature measured by an ear thermometer, and any recent onset of urinary tract symptoms. A dementia diagnosis required a comprehensive anamnesis and medical examination, laboratory tests, cognitive function test, and frequently neuroimaging.

### Laboratory tests

#### Concentrations of 25OHD

A nurse gathered blood samples from participants, which were analysed at Södra Älvsborg Hospital in Borås, Sweden, according to their established procedures. The samples were chilled prior to transport arriving for analysis within 24 h. Serum concentrations of 25OHD were analyzed by the LIAISON® 25 OH Vitamin D TOTAL Assay (DiaSorin Inc., Stillwater, USA) using chemiluminescent immunoassay (CLIA) technology for the quantitative determination of 25-hydroxyvitamin D. The range of the Assay was 4.0–150 ng/mL. This analysis was accredited at Södra Älvsborg Hospital.

#### Urine culture

Nursing staff members gathered a mid-stream morning urine sample, or a voided urine specimen with the longest possible bladder incubation time. The specimens were chilled prior to transport arriving at the laboratory within a day. The specimens were then cultured at Södra Älvsborg Hospital according to their established procedures using the outcomes of the dipstick urinalyses and information regarding UTI symptoms. The technical procedure is described in detail in a previously published study of bacteriuria and interleukin-6 concentrations, were data was collected together with this study [25]. Dipstick urinalyses and visual readings of the urine dipstick Multistix 5 (Siemens Healthcare Laboratory Diagnostics) were carried out at the nursing home. All urine specimens were cultured regardless of the outcome of dipstick urinalysis.

### Statistical analysis

The population was described according to the number of individuals, age, gender, 25OHD-concentrations, bacterial findings in urine cultures, vitamin D supplementation, and dementia or diabetes.

One objective was to describe vitamin D concentrations among residents with or without bacteriuria. Concentrations of 25OHD were compared between residents with positive and negative urine cultures using Student’s t-test. The proportion of residents with vitamin D deficiency defined by four cut-off concentrations was presented for all residents and differentiated by bacteriuria or not: 25OHD < 12.5 nmol/L, < 25 nmol/L, < 50 nmol/L and < 75 nmol/L.

Another objective was to establish whether bacteriuria was associated with vitamin D deficiency defined as 25OHD < 25 nmol/L and 25OHD < 12.5 nmol/L respectively, adjusted for age, gender, dementia and diabetes. Both adjusted and unadjusted logistic regressions were carried out.

All analyses employed the IBM SPSS Statistics version 22 (IBM Corporation, Armonk, New York, USA) and *p* < 0.05 was considered statistically significant.

## Results

### Study population

**Fig. 1:**
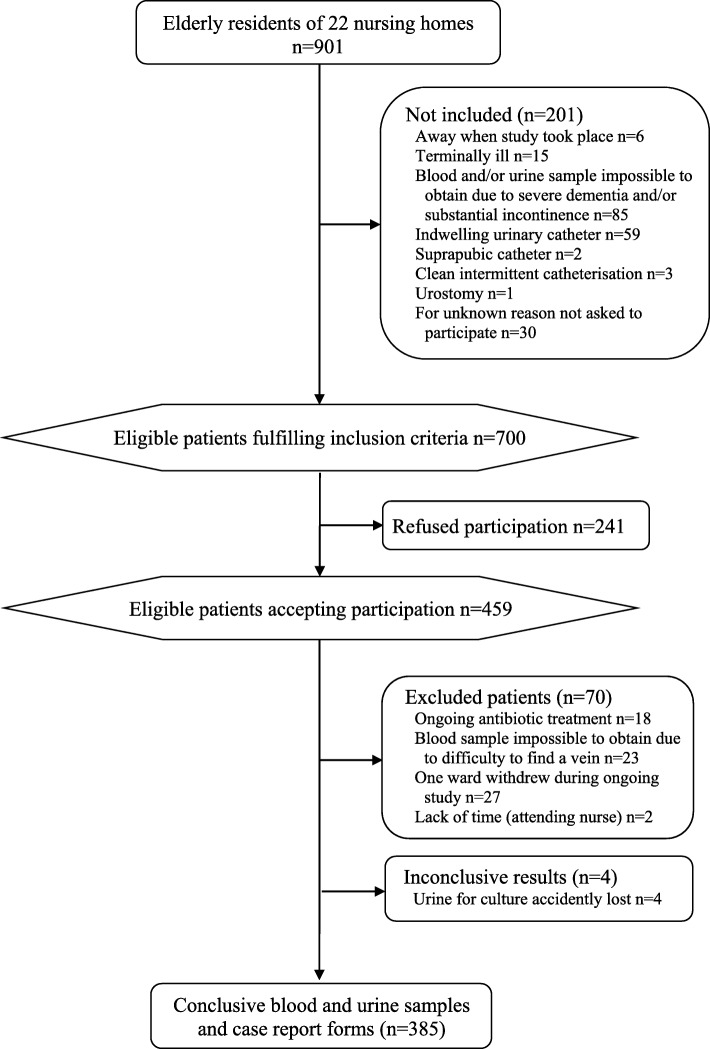
Participant flow chart

Seven hundred of nine hundred-one residents in 22 nursing homes met the inclusion criteria, and 459/700 (66%) agreed to participate (Fig. 1). 385 residents, 266 (69%) women and 119 (31%) men provided urine specimens, blood samples and case report forms. The mean age was 87 years-old (SD 6.7), and the range 63–100 with women (mean 87, SD 6.5, range 63–99) somewhat older than men (mean 85, SD 6.9, range 69–100) (*p* = 0.0091).

Fifthy-four percent (208/385) of participating residents had dementia, and 13% (51/385) diabetes (Table 1). Vitamin D supplementation was noted in 19% (73/385), and 82% (60/73) had colecalciferol (vitamin D_3_), and 18% (13/73) ergocalciferol (vitamin D_2_).

**Table 1:** Demographics and serum 25-hydroxyvitamin D (25OHD) concentrations among nursing home residents

	All residents^1^	Residents without bacteriuria	Residents with bacteriuria
Mean age (SD)	87 (6.7)	86 (6.9)	87 (5.9)
Women	69% (266/385)	58% (153/262)	92% (113/123)
Dementia	54% (208/385)	49% (129/262)	64% (79/123)
Diabetes mellitus	13% (51/385)	10% (27/262)	20% (24/123)
Dysuria, urinary urgency or frequency ≤ 1 week	1.6% (6/385)	1.5% (4/262)	1.6% (2/123)
25OHD < 12.5 nmol/L	3.1% (12/385)	1.9% (5/262)	5.7% (7/123)
25OHD 12.5 to < 25 nmol/L	34% (131/385)	33% (86/262)	37% (45/123)
25OHD 25 to < 50 nmol/L	46% (176/385)	48% (126/262)	41% (50/123)
25OHD 50 to < 75 nmol/L	10% (40/385)	11% (29/262)	8.9% (11/123)
25OHD > 75 nmol/L	6.8% (26/385)	6.1% (16/262)	8.1% (10/123)

^1^In total 385 residents: 262 residents without bacteriuria and 123 residents with bacteriuria

Prevalence of newly onset symptoms from the urinary tract during the last week was dysuria 0.78% (3/385), urinary urgency 1.3% (5/385), and urinary frequency 0.52% (2/385). All participants had a temperature < 38 °C.

### Serum 25OHD concentrations

Serum 25OHD concentrations, irrespective of vitamin D supplementation: mean 35 nmol/L (SD 21, median 28, range 4–125), 3.1% (12/385) < 12.5 nmol/L and 37% (143/385) < 25 nmol/L. Concentrations of serum 25OHD are presented in Table 1.

### Bacterial findings

Significant growth of potentially pathogenic bacteria was seen in 32% (123/385) of urine cultures. *E. coli* was present in 83% (102/123), the most common bacterial finding in positive urine cultures, the second most common finding was *Klebsiella* spp., present in 8.1% (10/123), and *Proteus* spp. were present in 1.6% (2/123). Other species had low prevalence, ≤ 1.6% for each species.

### Bacterial findings among residents with vitamin D deficiency

Among residents with 25OHD < 25 nmol/L 36% (52/143) had bacteriuria (Table 1). In those with < 12.5 nmol/L, 58% (7/12) had bacteriuria (Table 1). There was no difference in vitamin D concentrations among residents with or without bacteriuria (*p* = 0.76): mean concentrations of vitamin D were 34 nmol/L (SD 22) among residents with bacteriuria versus 35 nmol/L (SD 20) in those without bacteriuria.

### Factors associated with bacteriuria with cut-off 25OHD < 25 nmol/L

Adjusted OR (95% CI; *p*-value) for possible predictors of bacteriuria: 25OHD < 25 nmol/L 1.4 (0.86–2.3; *p* = 0.18), age 1.0 (1.0–1.1; *p* = 0.070), gender 7.5 (3.7–15; *p* < 0.001), dementia 1.9 (1.2–3.1; *p* = 0.012) and diabetes 2.3 (1.2–4.5; *p* = 0.014) (Table 2).

**Table 2:** Factors associated with bacteriuria, cut-off 25OHD^1^ < 25 nmol/L

	Unadjusted odds ratio^2^ (95% CI; *p*-value)	Adjusted odds ratio^3^ (95% CI; *p*-value)
25OHD < 25 nmol/L	1.4 (0.89–2.1; *p* = 0.15)	1.4 (0.86–2.3; *p* = 0.18)
Age	1.0 (1.0–1.1; *p* = 0.10)	1.0 (1.0–1.1; *p* = 0.070)
Gender^4^	8.1 (4.0–16; ***p*** **< 0.001**)	7.5 (3.7–15; ***p*** **< 0.001**)
Dementia	1.9 (1.2–2.9; ***p*** **= 0.0062**)	1.9 (1.2–3.1; ***p*** **= 0.012**)
Diabetes mellitus	2.1 (1.2–3.8; ***p*** **= 0.014**)	2.3 (1.2–4.5; ***p*** **= 0.014**)

^1^25-hydroxyvitamin D (25OHD)

^2^N = 385 included in analysis

^3^N = 385 included in analysis. Adjusted logistic regressions with bacteriuria as the dependent variable and the following independent variables: 25OHD < 25 nmol/L, age, gender, dementia and diabetes mellitus

^4^Reference category: male

Statistically significant findings are bold

### Factors associated with bacteriuria when using cut-off 25OHD < 12.5 nmol/L

Adjusted OR (95% CI; p-value) for possible predictors of bacteriuria: 25OHD < 12.5 nmol/L 4.4 (1.1–17; *p* = 0.031), age 1.0 (1.0–1.1; *p* = 0.060), gender 7.7 (3.8–16; p < 0.001), dementia 1.9 (1.2–3.2; *p* = 0.010) and diabetes 2.4 (1.2–4.7; p = 0.010) (Table 3).

**Table 3:** Factors associated with bacteriuria, cut-off 25OHD^1^ < 12.5 nmol/L

	Unadjusted odds ratio^2^ (95% CI; p-value)	Adjusted odds ratio^3^ (95% CI; p-value)
25OHD < 12.5 nmol/L	3.1 (0.96–10; *p* = 0.058)	4.4 (1.1–17; ***p*** **= 0.031**)
Age	1.0 (1.0–1.1; *p* = 0.10)	1.0 (1.0–1.1; *p* = 0.060)
Gender^4^	8.1 (4.0–16; ***p*** **< 0.001**)	7.7 (3.8–16; ***p*** **< 0.001**)
Dementia	1.9 (1.2–2.9; ***p*** **= 0.0062**)	1.9 (1.2–3.2; ***p*** **= 0.010**)
Diabetes mellitus	2.1 (1.2–3.8; ***p*** **= 0.014**)	2.4 (1.2–4.7; ***p*** **= 0.010**)

^1^25-hydroxyvitamin D (25OHD)

^2^N = 385 included in analysis

^3^N = 385 included in analysis. Adjusted logistic regressions with bacteriuria as the dependent variable and the following independent variables: 25OHD < 12.5 nmol/L, age, gender, dementia and diabetes mellitus

^4^Reference category: male

Statistically significant findings are bold

## Discussion

Both bacteriuria and vitamin D deficiency were common among the nursing home residents. No association was seen between bacteriuria and 25OHD < 25 nmol/L. There was an association if using < 12.5 nmol/L as a cut-off for vitamin D deficiency. However, this finding should be interpreted with caution whereby only a few residents had < 12.5 nmol/L.

### Strengths and limitations

That urine specimens and blood samples were collected from each resident accepting participation and of whom it was possible to get a voided urine specimen and a blood sample from January to March can be considered a strength of this study. Voided urine specimens, blood samples and study protocols were obtained from 43% (385/901) of the residents. Although not large due to dementia and urinary incontinence, this study visited 22 nursing homes and included a similar number of participants as previous studies [1, 6].

Another strength of this study is the adjustments for dementia and diabetes, well known confounders. Vitamin D deficiency and bacteriuria are more common among elderly residents at nursing homes with dementia, compared to elderly without [5, 27]. Also, bacteriuria and UTI are more common in diabetics [28, 29]. Despite adjusting for these well-known confounders there remains a risk for residual confounding and future research could further consider confounders related to frailty and multimorbidity. There were more women in this study, reflecting gender distribution in nursing homes. We also adjusted for gender in the logistic regressions.

A limitation of this study is that we observed bacteriuria and not UTI, so the clinical relevance of any associations is uncertain. Only a few residents in this study had newly onset symptoms from the urinary tract. Thus, most of the bacteriuric participants had ASB, not UTI. ASB causes a low grade inflammation of the epithelium in the urinary tract, and previous studies have shown an increased frequency of symptomatic UTI in those earlier identified with ASB [22–24]. Due to the increased risk of symptomatic UTI in patients with ASB it is important to study factors potentially associated with ASB. Thus, it may be relevant to study the association between ASB and Vitamin D deficiency.

There was an association between severe vitamin D deficiency (25OHD < 12.5 nmol/L) and bacteriuria. However, there were only 12 residents in this group. Due to small numbers, this association should be interpreted with caution. When elderly residents become frailer and their general health declines the frequency of ASB increases [30]. They are also more likely to be prone to severe vitamin D deficiency [31]. We studied the characteristics of these 12 residents to see if there was something that distinguished this group from the other residents: age, gender, dementia or diabetes. In this group 75% (9/12) had dementia compared to 53% (199/373) among those with 25OHD > 12.5 nmol/L. However, this was not a statistically significant difference (*p* = 0.14), and dementia was adjusted for in the logistic regression. Regarding age, gender and diabetes there were no differences at all between those with 25OHD above or below 12.5 nmol/L. As this is a cross-sectional study it is not possible to evaluate if there is causality between 25OHD < 12.5 nmol/L and bacteriuria, or just an association. Since there were only a few patients with severe vitamin D deficiency it is necessary to evaluate a possible association in a larger study. This research question is strengthened by the effectiveness of vitamin D supplementation in respiratory tract infections; a systematic review and meta-analysis showed reduced risk of acute respiratory tract infections after vitamin D supplementation, especially in those with baseline 25OHD < 25 nmol/L [32]. If an association is confirmed, a randomised controlled trial needs to be carried out to see if substitution with vitamin D could decrease the frequency of ASB, and subsequently also symptomatic UTI, since it is more common in those with ASB. Due to the evolving threat of antibiotic resistance it is important, if possible, to find methods to decrease the number of UTI. Regardless of causality or not there are other reasons for vitamin D supplementation, such as preventing osteoporosis and other conditions, in residents with 25OHD < 12.5 nmol/L [32–34].

### Statistical analysis

There is no present consensus for optimum serum concentrations of vitamin D [10, 11]. We chose to calculate the prevalence of vitamin D deficiency by four cut-off values suggested by previous studies and Swedish guidelines: 25OHD < 12.5 nmol/L, < 25 nmol/L, < 50 nmol/L and < 75 nmol/L [5, 35–37]. In the logistic regressions we used 25OHD as a dichotomized variable instead of 25OHD as a continuous variable: 25OHD < 25 nmol/L representing moderate vitamin D deficiency and < 12.5 nmol/L representing severe vitamin D deficiency [5, 35, 36]. This approach was chosen, as we wanted to see if a significant vitamin D deficiency was associated with bacteriuria, without being affected by different concentrations within vitamin D sufficiency. These cut-off values were pre planned in the statistical analysis. However, as a post hoc analysis we also performed a logistic regression using vitamin D as a continuous variable, and there was no association with bacteriuria (*p* = 0.47). In this analysis we adjusted for the same co-variates as when using 25OHD as a dichotomized variable: age, gender, dementia and diabetes mellitus.

## Conclusions

Bacteriuria was common among residents as well as vitamin D deficiency. There was no association between bacteriuria and 25OHD < 25 nmol/L. If using < 12.5 nmol/L as a cut-off for vitamin D deficiency, an association was seen. However, this finding has to be interpreted with caution as causality cannot be evaluated as well as only a few residents had 25OHD < 12.5 nmol/L. This has to be evaluated in a future study including more residents with severe vitamin D deficiency.

## Data Availability

The data that supports the findings of this study is available from the corresponding author upon request.

## Abbreviations

25OHD: 25-hydroxyvitamin D
AMP: Antimicrobial peptides
ASB: Asymptomatic bacteriuria
CFU: Colony-forming units
CI: Confidence interval
CLED: Cystine-lactose-electrolyte deficient agar
*E. coli*: *Escherichia coli*
nmol/L: Nanomole/litre
OR: Odds ratio
SD: Standard deviation
spp.: Species
UTI: Urinary tract infection
